# Active Control of Acoustic Field-of-View in a Biosonar System

**DOI:** 10.1371/journal.pbio.1001150

**Published:** 2011-09-13

**Authors:** Yossi Yovel, Ben Falk, Cynthia F. Moss, Nachum Ulanovsky

**Affiliations:** 1 Department of Neurobiology, Weizmann Institute of Science, Rehovot, Israel; 2 Department of Psychology, Institute for Systems Research and Neuroscience and Cognitive Science Program, University of Maryland, College Park, Maryland, United States of America; University of Ottawa, Canada

## Abstract

Active-sensing systems abound in nature, but little is known about systematic strategies that are used by these systems to scan the environment. Here, we addressed this question by studying echolocating bats, animals that have the ability to point their biosonar beam to a confined region of space. We trained Egyptian fruit bats to land on a target, under conditions of varying levels of environmental complexity, and measured their echolocation and flight behavior. The bats modulated the intensity of their biosonar emissions, and the spatial region they sampled, in a task-dependant manner. We report here that Egyptian fruit bats selectively change the emission intensity and the angle between the beam axes of sequentially emitted clicks, according to the distance to the target, and depending on the level of environmental complexity. In so doing, they effectively adjusted the spatial sector sampled by a pair of clicks—the “field-of-view.” We suggest that the exact point within the beam that is directed towards an object (e.g., the beam's peak, maximal slope, etc.) is influenced by three competing task demands: detection, localization, and angular scanning—where the third factor is modulated by field-of-view. Our results suggest that lingual echolocation (based on tongue clicks) is in fact much more sophisticated than previously believed. They also reveal a new parameter under active control in animal sonar—the angle between consecutive beams. Our findings suggest that acoustic scanning of space by mammals is highly flexible and modulated much more selectively than previously recognized.

## Abstract 2

Echolocating bats can actively change the area scanned by their biosonar sensory system (“field of view”), and they do so according to the complexity of the environment and depending on the distance to the target.

## Author Summary

Most sensory systems have an active component, i.e. driven by an animal's behavior, which contributes directly to its perception. For example, eye movements are important for visual perception, sniffs are crucial for olfactory percepts, and finger movements for touch percepts. A classic example of an active-sensing system is bat echolocation, or biosonar. Echolocating bats actively emit the energy with which they probe their surroundings, and they can control many aspects of sensory acquisition, such as the temporal or spectral resolution of their signals. A key open question in bat echolocation concerns bats' ability to actively change the area scanned by their emitted beam. Here, we used a large microphone array to study the echolocation behavior of Egyptian fruit bats. We found that these bats apply a new strategy to alter the area scanned by their beam; specifically, bats changed their acoustic field-of-view by changing the direction of consecutively emitted beams. Importantly, they did so in an environment-dependent manner, increasing the scanned area more when there were more objects in their surroundings. They also increased their field-of-view when approaching a target. These findings provide the first example for active changes in sensing volume, which occur in response to changes in environmental complexity and target-distance, and they suggest that active sensing of space is more flexible than previously thought.

## Introduction

The importance of “active sensing,” by which an animal actively interacts with the environment to adaptively control the acquisition of sensory information, is fundamental to perception across sensory modalities [1]–[7]. Echolocating bats emit ultrasonic signals and analyze the returning echoes to perceive their surroundings. Bat echolocation, an active sensory system, enables an acoustic representation of the environment through precise control of outgoing sonar signals. Laryngeal bats control many aspects of their sensory acquisition: they determine the timing of acquisition and the information flow [8]–[11], they control the intensity of the emission as well as its direction [12]–[17], and they control the spectral and temporal resolution of the acquired data [18]–[23]. Another acoustic parameter potentially under active control by echolocating bats is the pattern of the sonar beam. It has been debated whether bats can actively adjust the width of the sonar beam in response to task conditions, but empirical studies have not yet adequately addressed this question. It seems likely that bats would benefit greatly from the ability to control the beam pattern. They could for instance narrow the beam in order to concentrate energy onto a certain object, or they could widen the beam to increase the size of the sector that is being scanned. Studying the bat's active control over the shape and directionality of sonar emissions is technically difficult because reconstruction of the beam pattern requires a large circumferential ultrasonic microphone array in a setting where a free-flying bat engages in sonar tasks. A recent study suggests that laryngeal echolocating bats can change the space covered by their beam through adjustments in their call spectrum [24]. Here, we aimed to examine a very different mechanism by which echolocating bats might control the effective space they scan, namely adjustments in the angle between sequentially emitted sonar clicks.

We studied this question in lingual echolocating bats. Lingual echolocation is exhibited by one family of fruit bats, *Rousettus*, and has been historically considered to be more rudimentary than laryngeal echolocation [25]. The primary reason behind this notion was that these bats were believed to have very little control over their sonar emissions. In contrast, we recently demonstrated that the lingual echolocator *Rousettus aegyptiacus* (Egyptian fruit bat) uses a sophisticated strategy for beam-steering: This bat emits sonar clicks in pairs, and it directs the maximum slope of each sonar beam towards the target, rather than directing the center of the beam, thereby optimizing stimulus localization in the horizontal plane [15]. Here, we further tested Egyptian fruit bats' active control over their echolocation-based sensory acquisition. To this end, we tracked the flight trajectories of Egyptian fruit bats in a large room, and recorded their echolocation behavior when performing a landing task under different levels of environmental complexity. We found that lingual echolocation allows much more selective control over sonar signal parameters than previously believed. We discovered that Egyptian fruit bats alter the intensity of their emissions as they approach and lock the sonar beam onto a target, and that emission intensity changes with environmental complexity. Moreover, we found that Egyptian fruit bats apply a novel strategy to change the spatial region, or “field-of-view” that they scan: They increase the angle between the beam axes of sonar click-pairs, to effectively increase spatial scanning. Such a strategy has never been observed before in any bat species, and therefore comprises a new dimension of active control in lingual bat echolocation.

## Results

### Changes in Inter-Click Angle Along the Approach to a Landing Sphere in an Empty Room

**Figure 1:**
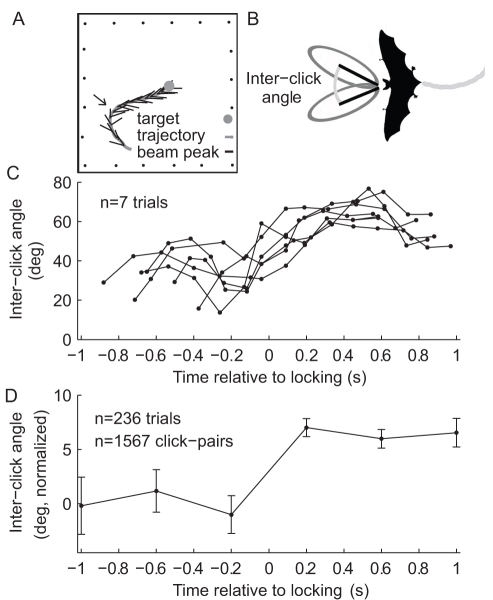
The inter-click beam angle is increased at the moment of locking when the bat is approaching a single object. (A) Schematic of single trial showing flight trajectory and direction of echolocation clicks in an Egyptian fruit bat (black lines). Dots at circumference, microphones; arrow, point of locking onto target. (B) Illustration of the inter-click angle. Black lines, direction of beam's peak; gray ellipse, polar representation of the sonar beam. (C) Examples of seven trials in which the inter-click angle abruptly increased around time 0 ( = the moment of locking). (D) Population average inter-click angle along the bats' approach to a single object. The angle was normalized separately for each bat to its average un-locked angle (see Materials and Methods). Error bars, mean ± s.e.m.; computed in 0.4-s bins.

In the first set of experiments—the “one-object experiments”—bats were trained to detect, localize, and land on a 10-cm diameter sphere, similar in size to fruit eaten by this bat species, such as mango. The sphere was the only object in an empty flight room (Figure 1A), and it was randomly moved between trials. Recordings were taken in complete darkness, forcing the bats to rely only on echolocation (see Materials and Methods). The echolocation of Egyptian fruit bats is comprised of pairs of clicks with a short inter-click time interval (∼20 ms) and a longer inter-pair interval (∼90 ms in complete darkness) [26],[27]. The bats direct their sonar beam axes Left-Right→Right-Left, maintaining a certain angle between the sequential clicks of a pair (Figure 1A–B) [15]. When approaching the target, bats significantly increased the inter-click angle by 6.8±0.4 degrees, on average (mean ± s.e.m.; *t* test of inter-click angle before locking versus after locking, when pooling all data together: *p*<10^−5^). This increase in inter-click angle occurred abruptly, coinciding with the time when the bats locked on the landing target, i.e. the time when the average direction of the click-pair coincided with the direction to the target (Figure 1C; see Materials and Methods) [15]. The increase occurred in all individual bats (Figure S1), and on average across all bats the change represented a 15% widening in the inter-click angle (post-locking compared to pre-locking). Population analysis of 236 trials (Figure 1D) confirmed that the increase of the inter-click angle was abrupt; in fact, it could occur within 2 click-pairs, i.e. as fast as 200 ms (Figure 1C–D).

This abrupt increase in inter-click angle may result from the bat's need to increase the field-of-view; or it may represent the animal's attempt to position the maximum slope of its sonar beam onto the target [15]. To further elucidate the possible roles of this abrupt change in inter-click angle, we conducted additional experiments that aimed to challenge the bat's scanning behavior. To this end, we manipulated the spatial complexity (number of objects) that the bat encountered within its field-of-view as it flew towards the landing sphere.

### Effects of Environmental Complexity on Inter-Click Angle

**Figure 2:**
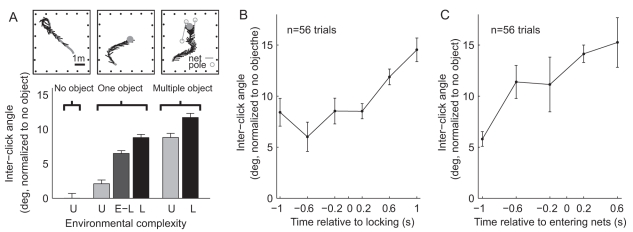
The inter-click angle increases with the increase in environmental complexity. (A) Top, schematic of the three experimental setups. Bottom, inter-click angle in the different experimental conditions. U, unlocked; L, locked; E-L, instances of early-locking prior to the final locking. Note increase in inter-click angle with environmental complexity. (B–C) Increase in inter-click angle along the approach, during multiple-object experiments. (B) In these experiments, the inter-click angle along the approach had a higher value (higher than in the one-object setup) and exhibited a gradual increase after the final locking onto the landing target. (C) When using the bat's entrance between the nets as an alternative locking criterion, it became evident that most of the increase in inter-click angle has occurred between 1 and 0.5 s before the bats entered in-between the nets. Note different *x*-axis in (B) and (C). Error bars, mean ± s.e.m.

In the next set of experiments, we manipulated the complexity of the environment, and examined how this influenced the Egyptian fruit bat's echolocation behavior. We hypothesized that when introducing a set of objects (obstacles) in the vicinity of the landing-point, which increases the environmental complexity, the bats would alter their scanning behavior to inspect several objects—thus increasing their field-of-view. To test this hypothesis, we studied the bats' behavior in two new setups (Figure 2A): (i) Open room condition: In 56 trials (8–12 trials per bat) we removed the sphere where the bats were trained to land. These trials were randomly introduced in between one-object trials; hence the bats reacted by vigorously searching for the target while flying around the room. We shall refer to this setup as the “no-object” experiment (Figure 2A, left). (ii) Environmentally complex condition: In 54 trials (8–11 per bat) we added two nets that were spread between four poles on both sides of the target, creating a relatively narrow (0.6–1.6 m) corridor for accessing the target (Figure 2A, right). The width of the corridor, its angle relative to the walls of the room, and the position of the landing sphere within the corridor were all randomly varied between trials. This setup mimics natural situations, in which a bat has to negotiate fruitless branches (the nets), before landing on a branch with a fruit (the target). We refer to this setup as the “multiple-object” experiment, because the bats consistently negotiated some or all of the five objects in the room—the single landing sphere (Figure 2A, right, closed gray circle) and the four poles (open circles). In all illustrations, bat's trajectory is depicted by a gray line and the direction of the beam's peak by a black line.

Egyptian fruit bats increased the angle between sequential clicks when environmental complexity increased (Figure 2A, bottom). The angular separation between the beam axes of sonar click pairs in the “no-object” setup was the narrowest; it increased in the one-object setup by 9.2±0.4 degrees (after locking, “L”), and increased even further in the multiple-object setup, widening on average by 12.3±0.6 degrees compared to the “no object” setup (Figure 2A bottom, “multiple object,” after locking). This behavioral pattern was consistent across all the individual bats that we tested (Figure S2). Statistical analysis showed that the increase in inter-click angle was highly significant (Figure 2A, bottom: one-way ANOVA: *F*>71, *p*<10^−8^; post-hoc *t* tests: *p*<10^−11^ for comparing one-object experiments after locking versus no-object experiments; *p*<10^−6^ for multiple-object experiments after locking versus one-object after locking). In the multiple-object setup, the bats increased the inter-click angle significantly beyond the point of maximum slope (i.e., the maximum slope of the beam was lateral to the target; *t* tests: *p*<10^−3^ for comparing one-object experiments after locking versus multiple-object experiments after locking). This suggests that, at least in this case, the inter-click angle plays another role in addition to placing the maximum slope on target for optimizing localization. We propose that widening the angle between the beam axes of sonar click pairs serves to modulate the bat's field-of-view. During the last time-bin before landing (Figure 2B, right-most point), the inter-click angle has increased on average by 14.5 degrees, compared to the mean angle in no-object experiments. When doing so, the point in the beam that was pointed to the center of the target was 2.5 degrees medial to the maximum slope. In the multiple-object experiment (Figure 2B), unlike in the one-object setup (Figure 1D), it seemed that the bats did not increase the inter-click angle abruptly (when we used the same locking criterion), but instead began the approach to the landing sphere with a large inter-click angle, and gradually increased even further after the final locking onto the landing target (Figure 2B). However, this gradual change may have been a result of temporal smearing that is specific to the multiple-object condition, and which is due to the difficulty in defining the exact time of “locking” in the multiple-object experiments: Although we defined sonar beam locking with reference to the landing sphere (i.e., when the average of the click pair was directed towards the landing target), the bats often locked onto the net's poles before locking onto the landing target (the 10-cm sphere). This means that they could have been in a “locked” sonar mode (locked onto a pole) when we defined them as un-locked relative to the landing target (see more details in the Discussion). We therefore tested an alternative sonar locking criterion for the multiple-object experiments, defining locking as the moment when the bats entered a corridor between the nets. This criterion revealed a clearer picture of the inter-click angle dynamics in the multiple-object situation (Figure 2C): Well before passing between the nets, the bats used an intermediate inter-click angle (5.8±0.7 degrees wider than no-object), which is between the locked and un-locked one-object situations. When the bats approached closer to the net corridor, they rapidly increased the inter-click angle to nearly its final value; subsequently, after the bats entered the net corridor, another slight increase was observed, which brought the inter-click angle to an average value that was 14.5±2.0 degrees wider than in the no-object experiments. At the plateau, the center of the target was ∼2.5 degrees beyond the maximum slope (*t* tests: *p*<10^−3^ for comparing one-object experiments after locking versus multiple-object experiments after locking). Maintaining such a high inter-click angle could possibly allow the bat to track *both* the target and the off-axis objects (distal poles) as the animal approaches landing—providing a potential strategy for target landing while avoiding collisions.

Interestingly, when further analyzing data from the one-object experiments, we found that in some trials, especially when the bats flew a long trajectory before landing, the bats sometimes locked their sonar on the landing sphere, then redirected the beam away and later performed the “final” locking when starting the final approach. The “E-L” bar (dark gray) in Figure 2A represents the inter-click angle during these “early locking” instances. It shows that the bats increased the inter-click angle even when they only transiently locked onto the target (during early locking, “E-L”). The widening of the inter-click angle in these instances was not as salient as in the final locking, probably because this beam-angle adjustment occurred for rather short periods of time (only a few click-pairs), and when the bats were rather far from the target (>1.5 m).

### Intensity Dynamics Along the Approach

**Figure 3:**
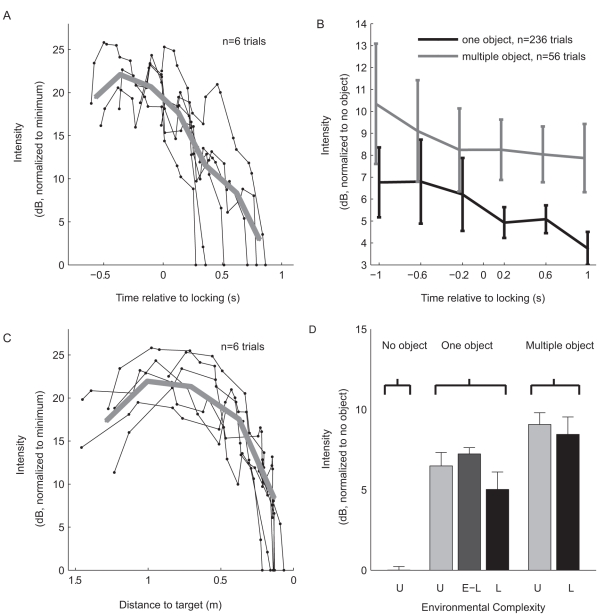
Lingual echolocators modify the intensity of their emissions according to the environmental complexity and the stage of target approach. (A) Examples of six trials, showing that emission intensity gradually decreases with time along the approach. Average is depicted by thick gray line. (B) Population average intensity plotted as function of time relative to locking, for the one-object and multiple-object setups. Error bars, mean ± s.e.m. The two curves were shifted by 30 ms relative to each other, for display purposes only. (C) Examples of the same six trials as in (A), with intensity plotted as function of distance to target. Average is depicted by thick gray line. Note decrease in intensity that began 80–100 cm before landing. (D) Click intensity increased with the environmental complexity. Intensity was not lower when the bats were locked on the target before the final locking (dark gray bar marked E-L).

In addition to the increase in inter-click angle, we found that Egyptian fruit bats decrease their emission intensity along the approach to landing (Figure 3A–C). We always refer here to peak intensity (see Materials and Methods), but since the duration of the sonar clicks is very constant, this is also highly correlated to the click's total energy. Because the bats in this experiment were free to choose the trajectory of landing, it was not always relevant to analyze the bat's distance to the target: for instance when a bat circles the target, it could be very close to it in terms of distance but very far in terms of time-to-landing (and may in fact be echolocating in a different direction). We therefore examined the intensity versus time-to-locking (Figure 3A–B), as well as intensity versus distance-to-target in trials in which the distance decreased nearly monotonically as the bat approached the landing sphere (Figure 3C). Figure 3C shows six examples in which the bat flew directly to the target, exhibiting a salient reduction in intensity, with a 4–6 dB decrease with halving of the distance-to-target during the final approach (Figure 3C, gray line, close to target). These results are consistent with reports in other bat species [28],[29]. Interestingly, this decrease in intensity began only 80–100 cm before landing—similar to what was observed in laryngeal echolocators [28]. Thus, the intensity dynamics along the approach seem to be shared by clicking and laryngeal bats.

### Changes in Click Intensity with Environmental Complexity

**Figure 4:**
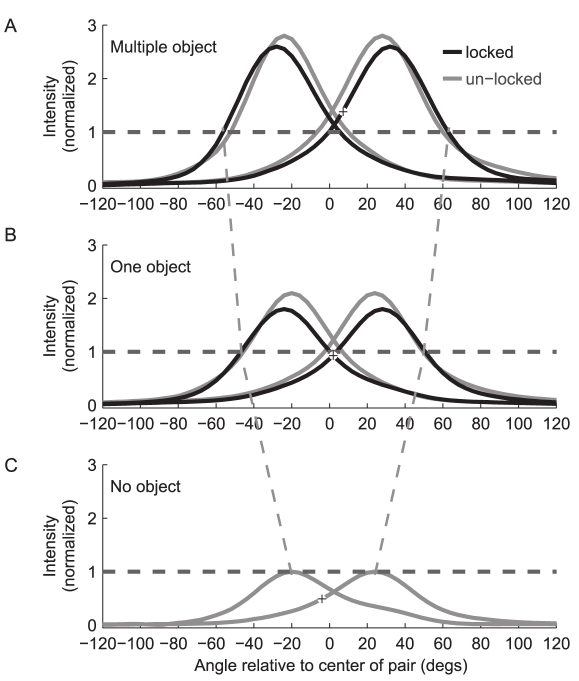
Combined effect of the changes in click intensity and changes in inter-click angle, across the different experimental setups. (A) Multiple-object experiment. (B) One-object experiment. (C) No-object experiment. Dashed gray lines depict the effective increase in field-of-view due to the combined increases in inter-click angle and click intensity; here we assumed a constant hearing threshold at normalized intensity of 1. Horizontal dashed lines, normalized intensity = 1; note that in all panels, this intensity was kept constant by the bats around their region of interest (see text). In all panels, “+” symbols depict the point of maximum-slope of the right beam (the locked beam when there are two): Note how the maximum slope changes position from being lateral (right) to the target in the multiple-object experiments, to pointing straight at the target in the one-object experiments, to being medial (left) to the target in the no-object experiments.

In addition to increasing the inter-click angle, bats also increased the intensity of their clicks with environmental complexity. The intensity increased by 6.5±0.6 dB on average in the one-object experiments compared with the no-object experiments, and further increased by 2.6±0.8 dB on average in the multiple-object experiments—that is, a total intensity increase of 9.1 dB in the multiple-object versus no-object condition (Figure 3D). These modulations of intensity could be used by the bat to maintain fixed signal energy directed towards the region of interest, compensating for changes in signal-to-noise ratio due to a widening field-of-view (see Figure 4, and next section). These differences in intensity were highly significant (one-way ANOVA: *F*>108, *p*<10^−9^; post-hoc *t* tests: *p*<10^−33^ for *t* test of one-object versus no-object; *p*<10^−16^ for multiple-object versus one-object; here we pooled together data from the approach phases before and after locking). Since we used a planar rather than a 3-D microphone array, and could not calculate the absolute emitted intensity, we performed explicit tests to control for the effects of bats' height, the distance from the microphones, and flight pitch (see Materials and Methods). The increase in intensity, together with the increase in inter-click angle, both contribute to an increase in the effective area that is sampled by the bats via a single click-pair (see next section and Discussion).

### Modulation of the Field-of-View

Our two main findings—that Egyptian fruit bats increase their inter-click angle and also increase the click intensity with increased environmental complexity—suggest that the field-of-view scanned by the bat is under active control and adapted to the environment. These adaptive sonar signal changes served to increase the bat's field-of-view when the environment became more complex (i.e., contained more objects). To examine this notion further, we quantified the field-of-view scanned by the bat, assuming a constant ensonification-intensity level and calculating the change in the angle of the sector covered by the bat's beam. When we used the intensity at the crossing point of the two beams in the one-object setup as reference (Figure 4 dashed lines, normalized intensity 1, see Materials and Methods), we found that the angle of the sector scanned by the bat with a single click-pair increased by a factor of 2.18 in the one-object experiments in comparison to the no-object (from 44 to 96 degrees), and by a factor of 2.73 in the multiple-object experiments in comparison to the no-object setup (from 44 to 120 degrees, see Figure 4C versus 4A). Interestingly, the same intensity (corresponding to a normalized intensity of 1 in Figure 4) is directed towards the crossing point of the two beams (where the object of interest is positioned) in both the multiple- and one-object setups, and it is the peak intensity (directed forwards) in the no-object setup. These modulations might thus reflect the bat's attempt to maintain a fixed energy impinging on the region of interest, compensating for the changes in signal-to-noise ratio due to the changes in field-of-view. The maximum distance (range) scanned by the bats also increased with environmental complexity, because detection range increases as the fourth root of the increase in intensity [30]. Thus, the 3-D “sensory volume” of space [31] that was scanned by the bats has increased at least 3-fold in the multiple-object versus the no-object experiment.

### Other Echolocation Parameters

We further examined several additional echolocation parameters in this set of experiments, and the results are summarized here. (i) We did not find any significant change in the beam width of the single clicks in the different environments. (ii) The bats did not significantly change the click repetition-rate in the multiple-object experiments in comparison to the one-object experiments (i.e., the intra-pair interval remained 23 ms on average and inter-pair interval was 93 ms on average). However, in the no-object experiments there was a small but significant decrease in the repetition rate, whereby the inter-pair interval increased by 8 ms (101 ms on average, *t* test of no-object versus one-object, for intervals >40 ms: *p*<10^−10^; Figure S3). (iii) We tested the spectral content of the echolocation clicks (recorded with a wide-band microphone) only in the one-object experiments; hence we cannot exclude changes in the spectra of the clicks. However, spectral changes seem physiologically unlikely, considering the tongue-production mechanism of the brief lingual clicks. Thus, the most salient changes that we observed were changes in inter-click angle, and changes in click intensity. These two parameters changed in opposite directions along the approach path to an object (inter-click angle increased while click intensity decreased during the approach), and both of these parameters increased substantially with environmental complexity.

## Discussion

The research findings presented here suggest that lingual (click-based) echolocation allows more adaptive control than previously reported. Egyptian fruit bats performing a landing task changed both their emission intensity and inter-click angle as they approached a target, in a manner that depended on both the environmental complexity and the behavioral phase. The increase in inter-click angle might serve two different functions: (i) *Pointing the maximum-slope to the target*: In the one-object setup, the increase in inter-click angle coincided with the moment of locking (Figure 1C–D), thus representing a behavioral phase-transition that could serve the function of directing the maximum slope to the center of the landing sphere, in order to optimize stimulus localization [15]. In comparison, in the no-object setup, the bats aimed most of the energy forward, in the direction of the flight, by decreasing the inter-click angle (Figure 2A, bottom). The narrow inter-click angle before locking, in the one-object situation, is very similar to the angle in the no-object situation, and might thus represent a narrow, forward focused field-of-view that is used before the final approach to the target. (ii) *Changing the field-of-view*: When shifting from the one-object to the multiple-object setup, the increase in inter-click angle was likely caused by the need to increase the field-of-view. In the multiple-object setup, the bats had to land on a specific target that was placed in the vicinity of other obstacles (e.g. poles, nets). In this situation, the bat's own motion created very large and rapid angular changes in the directions to nearby objects, and hence the bats would need to increase the field-of-view in order to track these objects.

Interestingly, the bats also decreased the emitted intensity while approaching the landing target (within a given level of environmental complexity). Such intensity decrease was not reported in a previous study of *Rousettus* echolocation [27], probably because they did not record bat signals during landing in that study. In our study, we observed a decrease in click intensity only during the last 80–100 cm before landing (Figure 3C), which suggests that the intensity decrease is initiated only when the bat actually approached the landing sphere. Thus, *Rousettus* bats increase the field-of-view and concurrently reduce the emitted intensity when approaching landing. A similar behavior is exhibited by approaching laryngeal echolocators [24]: The calls in the terminal group of these bats have more energy in low frequencies, and thus a wider beam, but also lower peak intensity. Lingual echolocators (e.g., Egyptian fruit bats) seem to have developed an alternative way to increase the effective beam width, which does not require them to change the spectral content of their emission. Instead, they change the scanning width by adjusting the angle between the axes of these two beams, and may treat the echoes returning from two consecutive clicks as a single “information unit” [15]. Such a strategy, which is based on adjusting the angular separation between two consecutive sonar emissions within a click-pair, has never been reported in any bat species to date, and it suggests an alternative adaptive mechanism in bat echolocation to sample a wider spatial region. Laryngeal echolocators are also known to steer their beams [13] and could thus also adjust the directional aim of successive sonar calls to control spatial sampling. However, there is no evidence for any laryngeal echolocator that constantly emits pairs of signals, similar to the Egyptian fruit bat; and accordingly, there is no evidence for any laryngeal echolocating bat that regards pairs of signals as their basic “sonar unit.” In addition, Egyptian fruit bats are probably able to achieve such quick changes in beam steering by rapid tongue movements [15], while changes in beam steering in laryngeal echolocators would probably require head movements, and would thus be slower than 20 ms. Thus, the field-of-view control strategy, suggested here for Egyptian fruit bats, might be a unique phenomenon among echolocating animals.

The increase in emission intensity in the different environmental setups may represent an attempt by the bat to maintain fixed energy directed towards the region of interest, thus compensating for changes in signal-to-noise ratio due to changes in field-of-view. Figure 4A–B shows that the region of interest (i.e., the crossing-point of the right and left beams) has approximately the same intensity in the one-object as in the multiple-object setups (horizontal dashed lines). Interestingly, the peak intensity that is being directed towards the direction of interest in the no-object setup is identical to the crossing-point-intensity in the one-object and multiple-object setups (Figure 4C, horizontal dashed line shows normalized intensity 1). This could be interpreted as a principle of “conservation of signal-to-noise” in lingual bat echolocation, and can explain the seemingly paradoxical behavior of decreasing emission intensity when performing a search task (in the no-object setup).

In a previous report [15], we described a trade-off between detection and localization in the Egyptian fruit bat, whereby detection is maximized by pointing the peak of the beam towards an object, while localization is optimized by pointing the maximum-slope towards the object. This tradeoff predicts that the bat will direct its sonar beam towards an object of interest at an angle that rests between the peak and the maximum slope. In our current multiple-object experiment, the bats deviated from this principle by consistently increasing the inter-click angle such that they directed the beam towards the target at points *beyond* the maximum slope of the beam (Figure 4A, see “+”). This finding is surprising, because it means that target localization cues were now likely diminished. In light of these results, as well as the other results presented in this article, we believe that a new dimension has to be considered, thus introducing a *three*-way tradeoff between (i) detection, (ii) localization, and (iii) angular scanning (modulated via changes in field-of-view). We suggest that in a complex environment, the need to scan the area around the landing-point, and to increase the field-of-view, is sufficiently important for the bats to reduce localization accuracy. Note that detection was actually *not* reduced by the increase in the inter-click angle in the more complex environments, because the bats also increased the click intensity, possibly as a compensatory mechanism (Figure 4A–C dashed lines).

What is the functional relevance of the sonar field-of-view? All the previous studies that were conducted on beam steering in laryngeal echolocating bats suggested that, despite their broad emission beams (60–70° width at −3 dB [24],[32]), these bats carefully direct the center of their beam towards the object of interest [13],[14],[24],[32]. Our previous study of sonar beam steering in Egyptian fruit bats showed that these lingual echolocators direct the center of their *beam*-*pair* onto the target [15], reminiscent of the individual calls of laryngeal echolocators. The behavior observed in the current study suggests that Egyptian fruit bats collect sensory information also from their acoustic periphery.

In the multiple-objects experiments, the bats exhibited a wide repertoire of behaviors before landing on the target (see details in Materials and Methods). In many cases (∼30% of trials) the Egyptian fruit bats only locked onto one of the poles, or occasionally did not lock on any of the poles while entering the corridor between the nets. We cannot completely exclude the possibility that the bats were relying on spatial memory (see Materials and Methods), but data from these trials imply that the bats can localize an object to some extent without the need to point the center of the beam-pair towards it. Increasing the field-of-view in order to follow objects near the landing target thus makes perfect sense from the bat's point of view.

In summary, our findings reveal two new aspects of adaptive control in lingual bat echolocation, namely the ability to change emission intensity as well as changing the inter-click angle between sequential emissions. The ability of lingual bats to change the inter-click angle reveals a new strategy for bats to actively control the field-of-view that they scan. Adjustment in field-of-view could also theoretically be exploited by laryngeal echolocators through movements of the head, mouth opening, and spectral changes in sonar emissions. The Egyptian fruit bat's directional aim of tongue click pairs demonstrates a new parameter of acoustic control in animal sonar. We suggest that environment-associated changes in emission intensity seem to be related to changes in field-of-view, and can compensate for decreases in signal-to-noise ratio due to changes in field-of-view. Further, our results suggest a three-way trade-off between three goals that a bat has to fulfill with its echolocation in a target-landing task: The *detection* of an object of interest, its accurate *localization*, and controlling the *field-of-view* that is being scanned by the bat. We believe that further studies of sensory trade-offs in echolocating bats will shed new light on bat echolocation—and more generally, on sensory constraints in active-sensing systems.

## Materials and Methods

### Training and Experiments

All experimental procedures were approved by the Institutional Animal Care and Use Committees of the Weizmann Institute of Science and the University of Maryland.

Five adult Egyptian fruit bats (*Rousettus aegyptiacus*) were trained to detect, localize, and approach a polystyrene sphere (10-cm diameter) that was mounted on a vertical pole positioned inside a large flight-room (6.4×6.4×2.7 m; Figure 1A). The target's size mimics the size of some fruits eaten by these bats in nature, such as mango. To minimize sound reverberations, the walls of the room were covered with acoustic foam and the pole was covered with felt. In order to ensure that the bats were relying solely on echolocation to perform the task, we took the following precautions: (i) To exclude the possibility of using visual cues, the target was painted black and the room was in complete darkness (illuminance <10^−4^ lux). The experimenter inside the room wore night-vision-goggles with infrared illumination. (ii) To prevent use of olfactory cues, the bats were food-rewarded only after landing on the target. The target was also cleaned with soap and water after every three trials to remove any possible odors that remained on it due to the contact with the bat. (iii) After every trial, the target was randomly re-positioned inside the room, both in the horizontal and in the vertical planes (the pole had a telescopic mechanism that allowed changing the target height). It took the bats ∼4 wk in order to learn the task and once they learned it they always succeeded in landing on the target.

### Environmental Complexity

The basic setting included only the landing target (10-cm polystyrene sphere) in the flight room. We also tested two alternative settings: (i) In 56 randomly interspersed trials we removed the landing target from the room, which made the bats eagerly fly in search for the target. We call these experiments the “no-object” experiments. (ii) In 54 trials, we added two nets mounted on 4 poles on both sides of the landing target (Figure 2A top, right). The distance between the nets randomly varied between trials (in the range of 0.6–1.6 m) and so did the position of the landing target and the angle of the nets in relation to the target. The bats learned to correctly land on the target between the nets—within 3–4 trials (which were not counted within these 54 trials); nevertheless, bats still occasionally landed on the poles even after many more trials. They were only rewarded for landing on the original target (sphere). Because these experiments involved five salient objects (1 target+4 poles), they were termed here “multiple-object” experiments.

The bats exhibited a wide behavioral repertoire in the multiple-object experiments: In some trials, they behaved similarly to the behavior described for the laryngeal echolocator, *Eptesicus fuscus* [14]. In those previous experiments, *E. fuscus* were trained to fly through a hole in a net, and they typically scanned both sides of the hole (pointing the peak of the beam to each edge of the hole) before flying through it. In the equivalent trials in the current study, Egyptian fruit bats locked the center of their click-pairs on both poles that outlined the opening of the net corridor, and only subsequently they flew through the corridor. In other trials, the Egyptian fruit bats either locked onto one of the poles before landing or did not lock on any of them. Because the bats had the opportunity to fly around the poles and nets before approaching them, and could thus learn their spatial locations, we could not completely exclude their relying on spatial memory. However, it is not likely that this was the only factor facilitating their approach, because the location and layout of the setup was always randomly changed between trials, and the bats did *not* always scan the setup before approaching the landing target.

The bat's average flight speed was negatively correlated with the environmental complexity (1.2±0.9 m/s in the multiple-object experiment, 1.9±1.2 m/s in the one-object experiment, and 2.4±1.0 m/s in the no-object experiment; mean ± s.d.; *p*<0.001 for all three *t* test comparisons, and *F*>880, *p*<10^−10^ in a one-way ANOVA test). This difference in flight speed remained when we analyzed the speeds only for pre-locking or only for post-locking epochs. We believe that the changes in flight speed were a result of the different maneuverability situation, due to the difference in the environmental complexity. In the multiple-object setup, the flight-speed likely decreased also because of the need to slow down in order to allow more time to scan the setup (in the multiple-object experiments, the bats typically slowed their flight before entering the net corridor, or when scanning the poles). Since the bats had the possibility to pre-scan the room, they could potentially adjust their speed to the expected maneuverability conditions, and this is likely why we did not see a change in flight speed between the pre-locked and post-locked situations.

### Sound Recordings

The bats' echolocation behavior was recorded with an array of 20 microphones spaced 1-m from each other around a rectangular supporting frame (5.3×5.2 m), at a height of 90 cm above the floor (Figures 1A and 2A, top: black dots around the circumference of the room show microphone locations) [32]. The signal from each microphone was amplified and fed into a band-pass filter centered around 35 kHz, with a frequency response that matches the frequency content of the *Rousettus* sonar click (see details in ref. [15]). Next, the signal was fed to an electronic circuit which extracted the envelope of this band-passed signal. The envelope was then low-pass filtered and digitized into a data-acquisition computer. Finally, the maximum value of this signal was translated into a dB scale in which analysis was performed. In order to control for changes in click spectra, in ∼20 trials of the one-object experiment we have recorded the audio using three wideband ultrasonic microphones positioned on the floor (sampled at 250 kHz/channel).

### Inclusion Criteria for Sonar Clicks

To ensure that we were only using high-quality data, we included only clicks that were clearly above noise level in at least five microphones of the array. In addition, we excluded beam measurements that were either too wide or too narrow relative to the overall distribution of >5,000 beam patterns recorded during >300 trials, because deviant widths led us to suspect a recording artifact due to temporary noise in some of the channels. To this end, we measured the width of the beams [15], and accepted only clicks with: 30°<*beam width*<120°. This resulted in exclusion of ∼6% of the clicks. In total, we analyzed here 5,144 sonar clicks from 346 behavioral trials in 5 bats (56 no-object trials, 236 one-object trials, and 54 multiple-object trials). We only analyzed clicks that occurred more than 250 ms before landing, because later clicks were emitted when the bat was too close to the target (closer than 15 cm on average), where any angular calculation of direction-to-target would suffer from very high error. This typically corresponded to excluding the last two click-pairs in the trial.

### Calculation of Inter-Click Angle and Click Intensity

All 20 signals (from 20 microphones) were first segmented to include vocalizations and exclude echoes. Then, the intensity at each microphone was corrected for spherical loss and atmospheric attenuation according to the measured position of the bat and the temperature and humidity in the flight room [32]. The *click intensity* was then taken as the maximum of these 20 intensity values. In order to calculate the beam direction, we averaged the direction of all microphones that recorded intensities of at least 0.8 of the maximum intensity or higher. This was done after smoothing the raw beam intensities with a 3^rd^-degree Golay-Savitzky filter [15]. Taking into account the system's noise and our beam estimation method, the error in beam-direction estimate was ∼5.5° (see ref. [15]). The *inter-click angle* was taken as the difference between two consecutive beam directions within a pair of clicks. The pairs are easy to recognize and can be mathematically defined as two clicks with a time-interval of less than 35 ms between them (Figure S3).

### Video Recording

Two high-speed digital video cameras (Photron, set with a frame rate of 125 frames per second), synchronized with the ultrasonic array, were used to record the flight of the bats. The direct-linear-transform algorithm was used to measure the three-dimensional location of the bat and other objects in the room, using the two camera views.

### Locking Criterion

We defined a “locked” click-pair as a pair in which the vector-average direction of its two clicks was <30° relative to the target (see example in Figure 1A; locking time is denoted by arrow). The 30° criterion was chosen since it corresponds to twice the asymptotic standard deviation of all click-pair vector averages, just before landing [15]. This is the same locking criterion as used in our previous study [15]. We tested two additional criteria for locking threshold (20° and 40°, unpublished data), which did not affect the results.

### Controls

Because our microphone-array was planar, we could not estimate the absolute emission intensity (sound pressure level). In order to be sure that the differences we found in the emitted intensity were not a result of some recording artifact, we tested whether the measured intensity of echolocation clicks is correlated with several flight-trajectory parameters: (i) distance from microphones (*r* = −0.03; n.s.); (ii) height of flight (*r* = 0.02; n.s.); (iii) flight pitch (*r* = 0.06; n.s.). None of these parameters showed any correlation with the emission intensity. We could not control for the head's pitch angle, but an examination of the raw videos did not reveal any tendency of the bats to systematically change head pitch in an environment-dependent manner.

The sensitivity of the array could not have changed between setups because the multiple-object and the no-object experiments were interspersed in time between the one-object experiments.

To control for possible sound-occlusion effects due to the specific layout of objects in the room (e.g., the target may have blocked a specific microphone and thus may have artificially enlarged the measured inter-click angle), we re-ran the entire analysis, taking for the direction of the beam the direction of the single microphone that recorded the peak intensity (rather than weighing over several microphones). This analysis did not affect our findings. It should be noted that such an artifact is not likely for other reasons as well: (i) If the angle increase was a result of an “occlusion artifact,” the angle should have increased gradually (rather than abruptly) in the one-object experiments. (ii) If it were an artifact, we would not have observed a widening of the angle when the bat was far from the target in the pre-locked situations (“E-L” bar in Figure 2A, bottom).

In order to verify that the nets were not blocking sound waves and possibly causing some acoustic artifacts, we estimated the attenuation caused by the nets, by comparing the emission recorded from a test speaker without nets to that recorded through the nets; no difference was found for an impinging angle of 90° (i.e., when emission was perpendicular to the nets).

### Normalization

Because each bat produced its individual typical emission intensity and unique inter-click angle, we always normalized data from each bat separately before averaging across all bats. This means that we first calculated the average (intensity or inter-click angle) in the no-object setup and then calculated the average change relative to this value in the different setups (one-object and multiple-object) or different behavioral phases (unlocked versus locked). We next calculated the average normalized change for all bats in each of the experimental paradigms. Unless stated otherwise, all the data were normalized in comparison to the one-object condition (rather than to no-object condition), because we had almost 5 times more data-trials for the one-object experiments, which provided us with a smooth, robust baseline to compare to.

## Supporting Information

Figure S1 The inter-click angle increases when locking onto the target, in all individual bats. “B,” before locking; “A,” after locking. Error bars, mean ± s.e.m. Note also that the basal angle differs systematically between individual bats. “*” significant difference; individual significance values: *p*<0.005; *p*<0.02; *p*<0.001; *p*<0.001; *p*<0.05, respectively, for the five bats.
(EPS)

Figure S2 The inter-click angle increases with the increase in environmental complexity, in all individual bats. Notice that the basal angle differs between individual bats. “0,” no object; “1,” one object; “5,” multiple-object (five-objects) experiments. Error bars, mean ± s.e.m.
(EPS)

Figure S3 The distribution of pulse intervals is bi-modal and is similar across the different experimental settings. The two peaks in the histogram represent the inter-pair intervals (right peak) and intra-pair intervals (left peak). In the no-object experiments (light gray), the inter-pair intervals were slightly higher than in the other setups, and increased from ∼90-ms to ∼100-ms.
(EPS)
